# First person – Chad VanSant-Webb and Jessye Castro

**DOI:** 10.1242/dmm.052864

**Published:** 2026-03-05

## Abstract

First Person is a series of interviews with the first authors of a selection of papers published in Disease Models & Mechanisms, helping researchers promote themselves alongside their papers. Chad VanSant-Webb and Jessye Castro are co-first authors on ‘ MicroRNA-21 promotes dysregulated lipid metabolism and hepatocellular carcinoma’, published in DMM. Chad is a Medical Scientist Training Program (MSTP) student in the lab of Dr Kimberley Evason at University of Utah, Salt Lake City, UT, USA, investigating inflammation. Jessye is a PhD student in the same lab, investigating cancer biomarkers that allow for early-stage diagnosis of cancer.


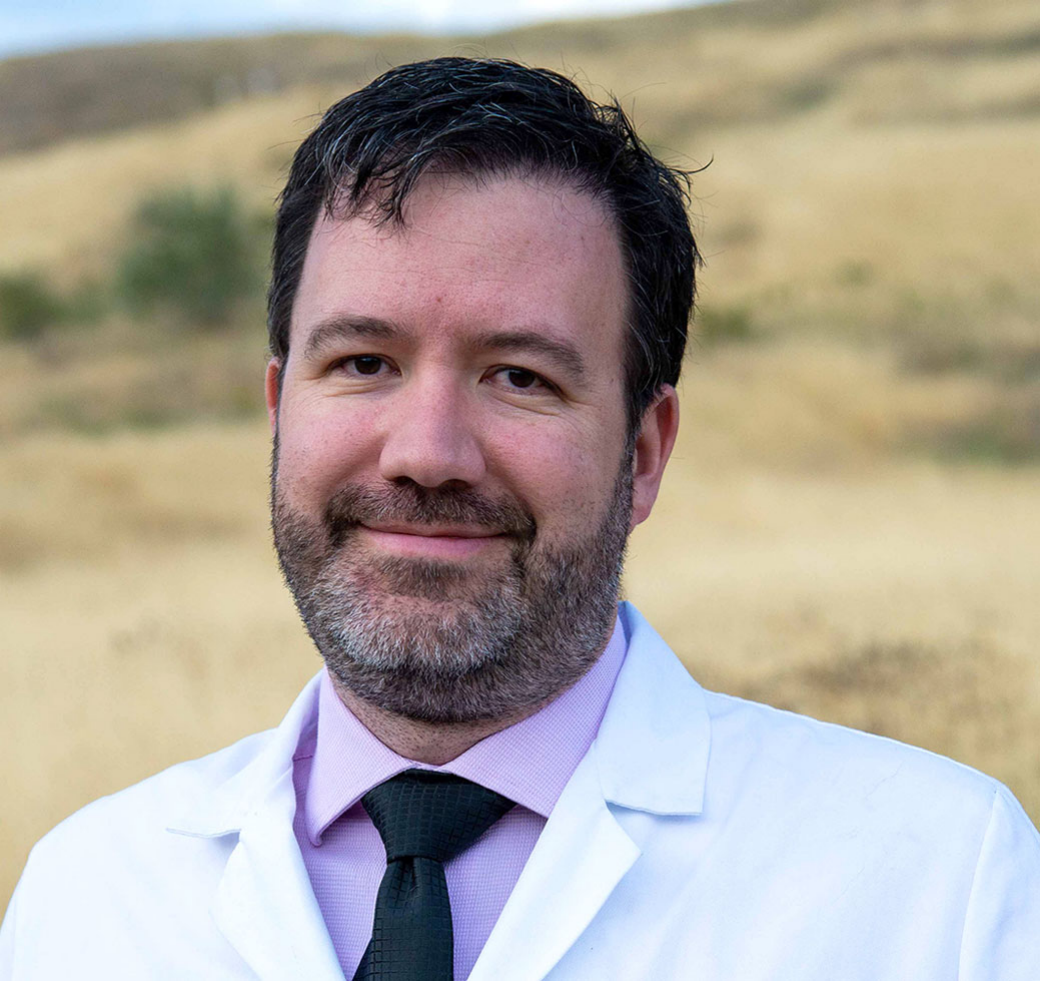


**Chad VanSant-Webb**

**Who or what inspired you to become a scientist?**

**C.V.-W.:** The scientific method is innate to the human experience, as babies and toddlers instinctively explore through experimentation. As a child one of my first words was ‘why’, and I am thankful that my parents and teachers fostered my curiosity and creativity. One person in particular is my uncle, who was a high-school science teacher and loved to support my curiosity most of all. While I enjoy learning for the sake of learning, I am fortunate to be able to combine my love for science and helping people as a physician-scientist.

**J.C.:** I can recall attending a lecture by Patricia Yew during my senior year of college. She was explaining her research on cell cycle events and how the mechanics differ in cancer. Until then, it hadn't occurred to me that molecular biology research could explain malignant cell growth – that malignancy could be studied. I thought there were medicine and clinical trials and then there was basic biology research. Dr Yew's talk opened my eyes to the field of translational research. I was immediately drawn to the career possibility of doing scientific discovery that can help patients. Since then, I have been pursuing projects that could forge a new therapeutic or diagnostic measure for specific cancers.

**What is the main question or challenge in disease biology you are addressing in this paper? How did you go about investigating your question or challenge?**

**C.V.-W.**/**J.C.:** There are many obstacles in researching and treating liver cancer, which tends to be diagnosed late in disease course. Recent advances in RNA-based discoveries and therapies provide opportunities to learn how liver cancer starts and progresses, with the hope of identifying potential therapeutics or interventions. Here, we used clinical samples to find changes to miRNAs as liver disease progressed, discovered that miR-21 is increasingly turned on as disease becomes more severe, and then used transgenic zebrafish to explore the mechanism of miR-21 dysregulation in disease pathology. Our findings provide insight into the functions of miR-21 in the liver and set up future experiments, which build on the strength of zebrafish to better understand the different roles that miR-21 plays in the context of liver metabolism, tumour initiation and progression.

**How would you explain the main findings of your paper to non-scientific family and friends?**

**C.V.-W.**/**J.C.:** A lot is known about how liver disease and cancer inappropriately turn genes ‘on’, but less is known about if and how genes are turned ‘off’. MicroRNAs act as a type of cellular thermostat to finely turn down the amount of specific genes. Our work shows that, during metabolic liver disease and liver cancer, cells turn specific genes ‘off’ by altering levels of microRNAs such as miR-21. Turning these genes ‘off’ changes how liver cells respond to fatty diets and stress. While this change can help liver cells survive in the short term, in the long term it may increase the risk of cancer.

… during metabolic liver disease and liver cancer, cells turn specific genes ‘off’ by altering levels of microRNAs such as miR-21

**What are the potential implications of these results for disease biology and the possible impact on patients?**

**C.V.-W./J.C.:** MiR-21 is well established as a cancer-promoting miRNA or ‘onco-mir’. Our work joins several other recent studies that bring nuance to this idea, by suggesting that there are benefits to hepatic miR-21 expression in the short term, such as increased steatosis clearance, but harmful effects in the long term, like increasing cancer risk. Our results show that elevated levels of fatty acid oxidation could provide a therapeutic target, but further work is needed to appreciate the context-specific differences of when and why fatty acid oxidation is increased during liver carcinogenesis and what is occurring downstream. Future studies may be able to identify miRNA-based treatments or molecular screening tools to understand how to screen, treat and/or monitor liver cancer.


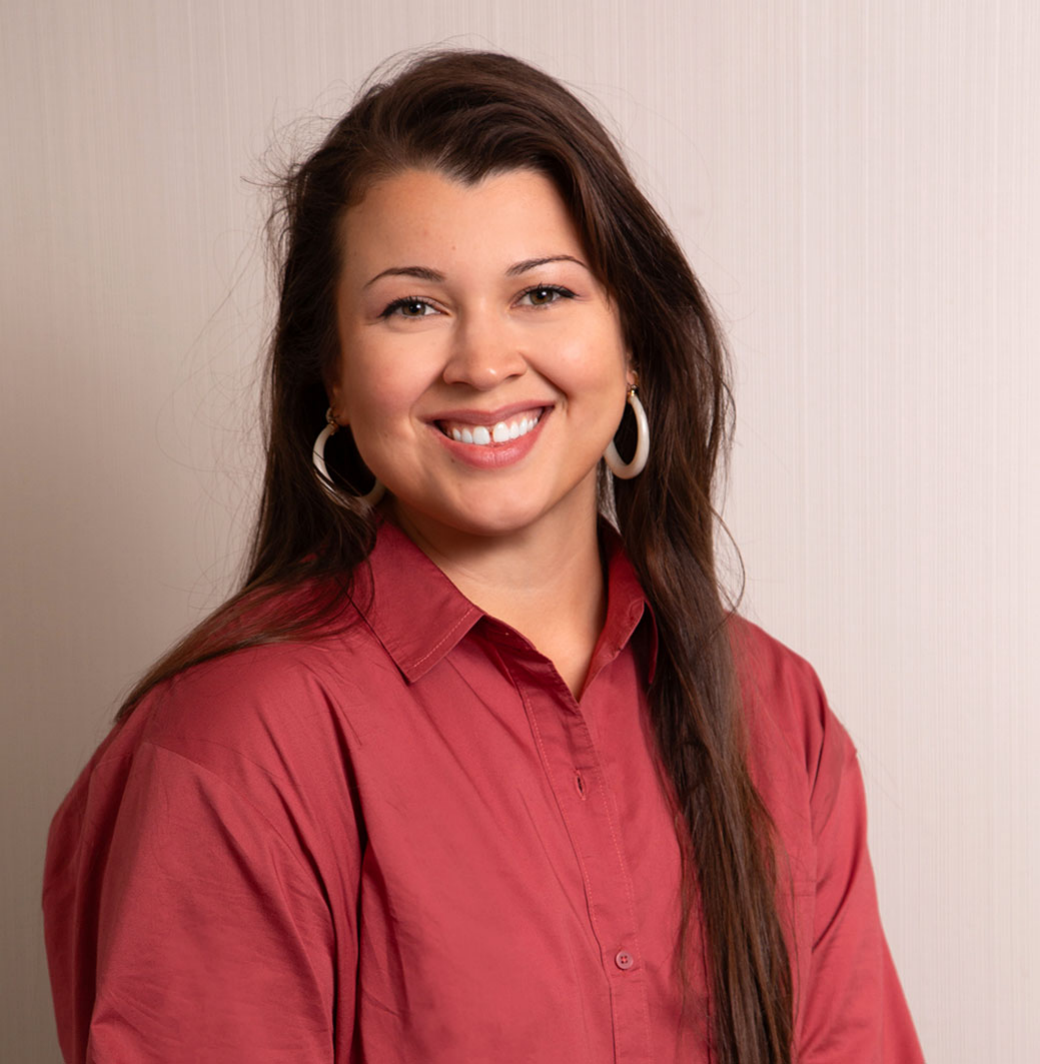


**Jessye Castro**

**Figure:**
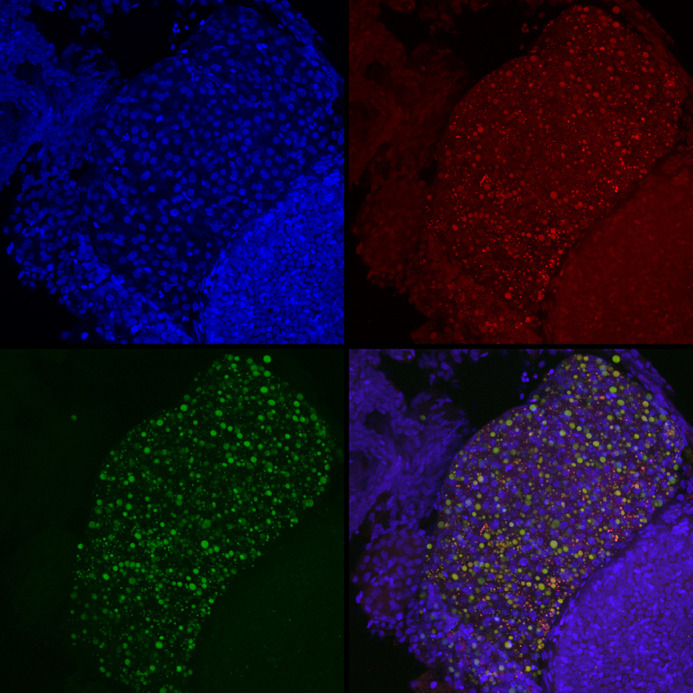
**Nile Red staining is a way to analyse lipid droplet composition and distribution.** We combined this stain with DAPI (blue) to evaluate changes to lipid droplets in hepatocytes following high-cholesterol diet. Composition of the lipid droplets can be visualized with the stain based on colour. Droplets containing phosphatidylcholine (PC) are visible in the red channel versus those containing neutral lipids (NL), shown in the green channel. The merged image in the bottom right shows how varied the droplet contents can be by the three colours of droplets: red (PC), yellow (both) and green (NL).

**Why did you choose DMM for your paper?**

**C.V.-W.**/**J.C.:** DMM has been one of our lab's favourite journals to read and reference, as it showcases strong scientific rigour while highlighting new and diverse scientific techniques and topics. DMM also has a strong focus on research that spans the basic and clinical spaces, so our work, which used zebrafish to model the miRNA dysregulation we identified in humans, felt like a perfect fit. It can be a shock for people to learn that not only do zebrafish have livers, which are very similar to human livers, but that they are effective model organisms to study liver physiology and disease. We also appreciate that DMM is open access to all.

… it is also up to politicians and society as a whole to take steps to value scientific education and research

**Given your current role, what challenges do you face and what changes could improve the professional lives of other scientists in this role?**

**C.V.-W.:** The post-COVID era has been an interesting time to be an RNA biologist, as scientific misinformation has been at an all-time high, particularly surrounding RNA. This has since led to increased instability of scientific funding, which, if uncorrected, will disrupt scientific progress for generations to come. While scientists can do more to engage with the public, it is also up to politicians and society as a whole to take steps to value scientific education and research.

**J.C.:** At the moment, there is a big push for AI and omics data integration into biomedical research. On many occasions during my PhD, I have felt the need to have better computer science skills and that I should be doing more rigorous omics experiments, especially as I begin my transition to industry. Access to advanced coding courses or workshops at the graduate level has helped me develop the skills to conduct and interpret biomedical data science. Continuing this access across institutions will equip graduate level scientists with a better toolkit for these change in biomedical research and will help prevent the pressure and anxiety of trying to acquire these skills on our own.

**What's next for you?**

**C.V.-W.:** I am excited to wrap up my education and training with the University of Utah Spencer Fox Eccles School of Medicine MSTP program and look forward to learning which physician-scientist training programme I match into in March 2026.

**J.C.:** I will be finishing my PhD soon and applying for positions at companies working to discover biomarkers and develop testing to implement in the clinic. I am interested in discovery and R&D levels of industry to continue building my research and communication skills.

**Tell us something interesting about yourself that wouldn't be on your CV**

**C.V.-W.:** Prior to medical school I had grown my hair to shoulder length out of curiosity and two funny things happened: (1) I learned that with long hair and a short beard, my doppelgänger is Mexican singer Marco Antonio Solís; and (2) despite what you see in media, people do not often shave their head to support a loved one with cancer! I had a drastic hair cut when my dad started chemotherapy for leukaemia, going from shoulder-length hair (which I donated) to completely shaved. I don't know who was more surprised, my dad or his oncologists! Thankfully, my dad is doing well and has been in remission for almost a decade.

**J.C.:** I am a member of the local community garden. I look forward to spring when I can get my hands in the dirt and start growing my own food. My favourite things to grow are carrots, potatoes, cucumber, corn and peas. The process of planting, nurturing and caring for the plants comes very easily after years of following protocols and optimizing conditions at the bench. In a time when climate uncertainty is at its highest, the garden helps me decrease my carbon footprint and support native bee populations, as well as promote sustainable agriculture practices. Establishing this connection with the earth has become very important to me and has made me appreciate living in a place where this kind of resource is available. The community garden is organic and chemical pesticide free, meaning I get to experiment with ways to protect my garden from pests. One extreme method I have used was releasing almost 400 ladybugs to eat the tiny aphids occupying the garden.
